# Supplementary material for: Comparative genomics provides new insights into the diversity, physiology, and sexuality of the only industrially exploited tremellomycete: Phaffia rhodozyma
Source: BMC Genomics. 2016 Nov 9;17:901. doi: 10.1186/s12864-016-3244-7 (PMC5103461; doi:10.1186/s12864-016-3244-7)
Supplement: Additional file 6: — List of orphan genes with links to PFAM (related to Additional file 1: Table S1). (ZIP 1428 kb) [file 12864_2016_3244_MOESM6_ESM.zip › BLAST_HTML_FTR/G00333_P.html]

BLAST Search Results


```
BLASTP 2.2.27+


Reference:
Stephen F. Altschul, Thomas L. Madden, Alejandro A. Schäffer,
Jinghui Zhang, Zheng Zhang, Webb Miller, and David J. Lipman (1997),
"Gapped BLAST and PSI-BLAST: a new generation of protein database
search programs", Nucleic Acids Res. 25:3389-3402.


Reference for
composition-based statistics:
Alejandro A. Schäffer, L. Aravind, Thomas L. Madden, Sergei
Shavirin, John L. Spouge, Yuri I. Wolf, Eugene V. Koonin, and
Stephen F. Altschul (2001), "Improving the accuracy of PSI-BLAST
protein database searches with composition-based statistics and
other refinements", Nucleic Acids Res. 29:2994-3005.


Database: nr
           71,551,133 sequences; 26,053,659,533 total letters


Query= G00333_P

Length=243
                                                                      Score     E
Sequences producing significant alignments:                          (Bits)  Value

emb|CED83218.1|  hypothetical protein [Xanthophyllomyces dendrorh...   476    5e-168
emb|CED85041.1|  hypothetical protein [Xanthophyllomyces dendrorh...  47.0    0.006 
ref|WP_050486704.1|  hypothetical protein [Streptomyces sp. CNS654]   43.1    0.22  
emb|CCA20935.1|  conserved hypothetical protein [Albugo laibachii...  40.8    1.3   
gb|KIR59330.1|  hypothetical protein I314_04847 [Cryptococcus gat...  40.0    2.1   
gb|KIR45327.1|  hypothetical protein I312_05368 [Cryptococcus gat...  39.7    2.4   
gb|KDQ25737.1|  hypothetical protein PLEOSDRAFT_1106641 [Pleurotu...  38.1    6.1   


 >emb|CED83218.1| hypothetical protein [Xanthophyllomyces dendrorhous]
Length=242

 Score =  476 bits (1225),  Expect = 5e-168, Method: Compositional matrix adjust.
 Identities = 242/242 (100%), Positives = 242/242 (100%), Gaps = 0/242 (0%)

Query  1    MFLASFPVSKPASSASDSSRRSFKVASVEEVEEDNDRGRGRSDQRKPLRFFYPTVRQPVQ  60
            MFLASFPVSKPASSASDSSRRSFKVASVEEVEEDNDRGRGRSDQRKPLRFFYPTVRQPVQ
Sbjct  1    MFLASFPVSKPASSASDSSRRSFKVASVEEVEEDNDRGRGRSDQRKPLRFFYPTVRQPVQ  60

Query  61   SSPIQIAQVSARSAQPQGLVDMSIPPILSTSPPSTTYLDLTRLASASPSPSRSAGSDCEC  120
            SSPIQIAQVSARSAQPQGLVDMSIPPILSTSPPSTTYLDLTRLASASPSPSRSAGSDCEC
Sbjct  61   SSPIQIAQVSARSAQPQGLVDMSIPPILSTSPPSTTYLDLTRLASASPSPSRSAGSDCEC  120

Query  121  AECVERDELSSESEGENAFDEDYSDFDEDEGDLADEDEVPVQAWAKKGRSAEKTGKFISG  180
            AECVERDELSSESEGENAFDEDYSDFDEDEGDLADEDEVPVQAWAKKGRSAEKTGKFISG
Sbjct  121  AECVERDELSSESEGENAFDEDYSDFDEDEGDLADEDEVPVQAWAKKGRSAEKTGKFISG  180

Query  181  IAVPFSKTSIPTLTFARNPRSFPKSPAFSFSSSSSPSSSGVSTLLSMPPTNAPFLVGSPS  240
            IAVPFSKTSIPTLTFARNPRSFPKSPAFSFSSSSSPSSSGVSTLLSMPPTNAPFLVGSPS
Sbjct  181  IAVPFSKTSIPTLTFARNPRSFPKSPAFSFSSSSSPSSSGVSTLLSMPPTNAPFLVGSPS  240

Query  241  YR  242
            YR
Sbjct  241  YR  242


>emb|CED85041.1| hypothetical protein [Xanthophyllomyces dendrorhous]
Length=216

 Score = 47.0 bits (110),  Expect = 0.006, Method: Compositional matrix adjust.
 Identities = 24/55 (44%), Positives = 36/55 (65%), Gaps = 3/55 (5%)

Query  5   SFPVSKPASSASDSSRRSFKVAS-VEEVEEDNDRGRGRSDQRKPLRFFYPTVRQP  58
           + P+ +  + +SD+  RSF  A  V+   E+  RGRGRSD +KPLRF +PT ++P
Sbjct  21  ALPIVRIRTVSSDA--RSFDWAEDVDRYYENETRGRGRSDAKKPLRFLFPTAQEP  73


>ref|WP_050486704.1| hypothetical protein [Streptomyces sp. CNS654]
Length=3137

 Score = 43.1 bits (100),  Expect = 0.22, Method: Compositional matrix adjust.
 Identities = 40/164 (24%), Positives = 72/164 (44%), Gaps = 27/164 (16%)

Query  74    AQPQGLVDMSIPPILSTSPPSTTYLDLTRLASASPSPSRSAGSDCECAECVERDELSSES  133
             AQ +G+ D  +P I  T      ++D  RLA    S           AE + R  L   +
Sbjct  1542  AQEEGIEDADVPGIALTLQVGREHMD-ERLAFVVGS----------AAELMAR--LREHA  1588

Query  134   EGENAFDEDYSDFDEDEGD---LADEDEVP--VQAWAKKGRSAEKTGKFISGIAVPF---  185
             +G++  +         EG    LAD++++   ++AW +KG+  +    +++G+AVP+   
Sbjct  1589  DGKDGAEVFRGRIRRAEGAVSFLADDEDMAHTIEAWVRKGKFGKLLELWVNGLAVPWRRS  1648

Query  186   ------SKTSIPTLTFARNPRSFPKSPAFSFSSSSSPSSSGVST  223
                    + S+PT  FAR     P     + +S   P ++  +T
Sbjct  1649  YPDPLPDRVSLPTYPFARKRFWLPTGAPATVASDRRPQATATAT  1692


>emb|CCA20935.1| conserved hypothetical protein [Albugo laibachii Nc14]
Length=1172

 Score = 40.8 bits (94),  Expect = 1.3, Method: Compositional matrix adjust.
 Identities = 38/124 (31%), Positives = 53/124 (43%), Gaps = 19/124 (15%)

Query  48   LRFFYPTVRQPVQSSPIQIAQVSARSAQPQGLV---DMSIPPILSTSPPSTTYLDLTRLA  104
            L +  P + +  QS P   A+  A  A  + +     +SIPP+   SP ST Y +LT+L 
Sbjct  232  LLYLVPILLEVAQSDPCADAKFDALCALKELIAAFQHLSIPPV--QSPSSTLYRELTQLH  289

Query  105  SASPS--------------PSRSAGSDCECAECVERDELSSESEGENAFDEDYSDFDEDE  150
               P               PSR      EC   V R  LS  S  +++  + Y  FDE +
Sbjct  290  LIFPGIATGLWMAVDAPKRPSRVVSLAIECFADVARAVLSDSSLMKHSKKQLYHAFDEVK  349

Query  151  GDLA  154
            G LA
Sbjct  350  GILA  353


>gb|KIR59330.1| hypothetical protein I314_04847 [Cryptococcus gattii CA1873]
Length=754

 Score = 40.0 bits (92),  Expect = 2.1, Method: Compositional matrix adjust.
 Identities = 32/109 (29%), Positives = 49/109 (45%), Gaps = 9/109 (8%)

Query  131  SESEGENAFDEDYSDFDEDEGDLADEDEVPVQAWAKKGRSAEKTGKFISGIAVPFSKTSI  190
            S +E     D D  D D   G   D +++ +  W +  R  +K   FI+G  V   +   
Sbjct  35   STAELHEQLDTDLDDID---GWSEDSEDIEIYKWTRPLRVKKKKINFITGRVVTNPRHRR  91

Query  191  PTLTFARNPRSF----PKSPAFSFSSSSSPSS--SGVSTLLSMPPTNAP  233
             T++  R+PRSF    PKSP    S ++ P S  + +S L    P  +P
Sbjct  92   STISGPRSPRSFTSASPKSPTLERSCAAEPVSAHAHISQLFFALPATSP  140


>gb|KIR45327.1| hypothetical protein I312_05368 [Cryptococcus gattii CA1280]
Length=754

 Score = 39.7 bits (91),  Expect = 2.4, Method: Compositional matrix adjust.
 Identities = 32/109 (29%), Positives = 49/109 (45%), Gaps = 9/109 (8%)

Query  131  SESEGENAFDEDYSDFDEDEGDLADEDEVPVQAWAKKGRSAEKTGKFISGIAVPFSKTSI  190
            S +E     D D  D D   G   D +++ +  W +  R  +K   FI+G  V   +   
Sbjct  35   STAELHEQLDTDLDDID---GWSEDSEDIEIYKWTRPLRVKKKKINFITGRVVTNPRHRR  91

Query  191  PTLTFARNPRSF----PKSPAFSFSSSSSPSS--SGVSTLLSMPPTNAP  233
             T++  R+PRSF    PKSP    S ++ P S  + +S L    P  +P
Sbjct  92   STISGPRSPRSFTSASPKSPTLERSCAAEPVSAHAHISQLFFALPATSP  140


>gb|KDQ25737.1| hypothetical protein PLEOSDRAFT_1106641 [Pleurotus ostreatus 
PC15]
Length=287

 Score = 38.1 bits (87),  Expect = 6.1, Method: Compositional matrix adjust.
 Identities = 27/82 (33%), Positives = 39/82 (48%), Gaps = 3/82 (4%)

Query  80   VDMSIPP---ILSTSPPSTTYLDLTRLASASPSPSRSAGSDCECAECVERDELSSESEGE  136
            VD S PP   + +TS     Y+ + R      S S S GS   C E VE   L+++   +
Sbjct  159  VDGSPPPKFHLTATSNTGALYIHIPRSFHGPISASSSTGSVHLCRELVENATLTADGHTK  218

Query  137  NAFDEDYSDFDEDEGDLADEDE  158
              F  DY D D+D G+   ++E
Sbjct  219  KCFVGDYPDVDDDRGNDGRKNE  240


Lambda      K        H        a         alpha
   0.312    0.127    0.360    0.792     4.96 

Gapped
Lambda      K        H        a         alpha    sigma
   0.267   0.0410    0.140     1.90     42.6     43.6 

Effective search space used: 1513897829155


  Database: nr
    Posted date:  Sep 23, 2015 12:05 AM
  Number of letters in database: 26,053,659,533
  Number of sequences in database:  71,551,133


Matrix: BLOSUM62
Gap Penalties: Existence: 11, Extension: 1
Neighboring words threshold: 11
Window for multiple hits: 40
```
